# Fascicular Visual Field Defects in Open-Angle Glaucoma: Evaluation with Microperimetry

**DOI:** 10.1155/2016/8274954

**Published:** 2016-06-05

**Authors:** Loredana Arrico, Rossella Giannotti, Manuela Fratipietro, Romualdo Malagola

**Affiliations:** Department of Sense Organs, Sapienza University of Rome, Viale del Policlinico 155, 00161 Rome, Italy

## Abstract

*Purpose*. Use of microperimetry (Mp-1), correlating with Humphrey perimetry (30-2 program), in patients affected by primary open-angle glaucoma (POAG) with perimetric defects, in order to obtain an evaluation of the accuracy of the results obtained by Mp-1.* Materials and Methods*. In this study 40 eyes of 25 patients affected by POAG with perimetric defects were included. All patients underwent microperimetry test by Nidek Mp-1 (NAVIS software version 1.7.2, Nidek Technologies). Mean sensitivity values expressed in decibel (dB) of all tested dots and mean values for each quadrant obtained by microperimetric test were correlated with corresponding quadrants obtained by static perimetry analysis. Data were analyzed by Pearson's correlation and Bland-Altman analysis.* Results*. Interpolated data showed that mean sensitivity values in all spots tested by Mp-1 (11.98 dB, SD 4.31) may be significantly correlated with mean total values obtained by Humphrey 30-2 perimetry (17.95, SD 4.32), with correlation coefficient of 0.556.* Conclusions*. Topographic visualization of the perimetric alteration by microperimetry allows retesting areas with reduced sensitivity which are topographically visualized and displayable on the ocular fundus examination, avoiding worsening of the functional defect by better modulation of the antiglaucoma therapy and therefore it allows better monitoring of the pathologic functional damage.

## 1. Introduction

Glaucoma is classically recognized as the main ocular condition causing irreversible blindness occurring in 70 million subjects worldwide. It consists in an ocular neuropathy progressing into an over loss of retinal ganglion cells (RGC) and their axons which determines the crucial organic-functional damage [1].

For the diagnosis of glaucoma, the detection of alteration occurring at the optic nerve head level, abnormalities of the retinal nerve fiber layer (RNFL), visual field loss, and the presence of elevated intraocular pressure (IOP) is fundamental.

Currently, various assessment and functional tests are used for the diagnosis of the glaucoma (assessment: namely, scanning laser polarimetry, Heidelberg Retinal Tomography, and optical coherence tomography. Functional test: computerized automated perimetry: Humphrey, Octopus; color vision study; contrast sensitivity study; light accommodation study). Among functional tests, the automated (computerized) perimetry is considered the gold standard for both diagnosis and follow-up because of its elevated sensitivity to detect perimetric abnormalities using full threshold test or SITA-Standard with Humphrey perimeter evaluating a sufficient dot density, distributed within “the 30° area.”

Nevertheless, the computerized automated perimetry is not able to identify the area and quantify the stability of the fixation, particularly in partially sighted subjects. The fundamental issue is the impossibility of controlling ocular movements and displaying ocular fundus in real time during the test.

Conversely, microperimetry (MP) is a new-generation morphofunctional technique that does not show this limit, allowing performing an electronic view field contemporarily to the display of stimulated dots within patient's ocular fundus.

**Figure 1:**
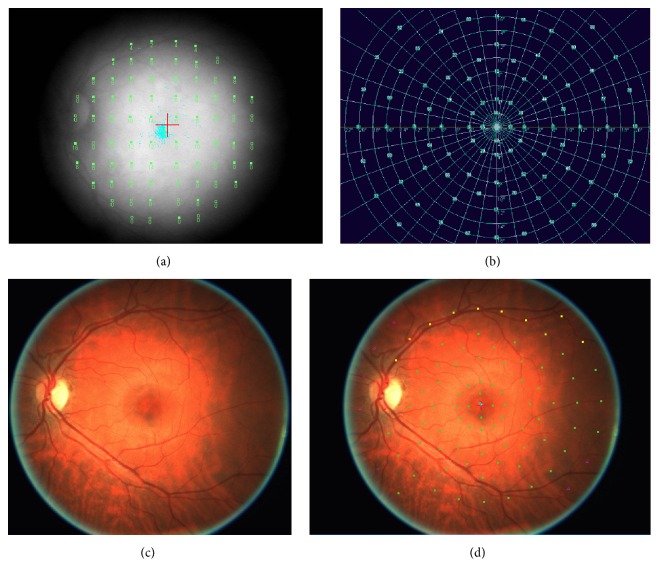
(a) Infrared image of the ocular fundi. (b) Computerized perimetry. (c) Digital image of the ocular fundi. (d) Microperimetric image.

The sensitivity map is generated by observing in a continuative way the ocular fundus and projecting light stimuli in retinal areas selected by the operator. In this way, it becomes possible to correlate specific dots within the ocular fundi with the eventual alterations of retinal sensitivity and thus to associate objectively the morphologic retinal changes ophthalmoscopically observed and the subsequent functional alterations (Figure 1).

**Figure 2:**
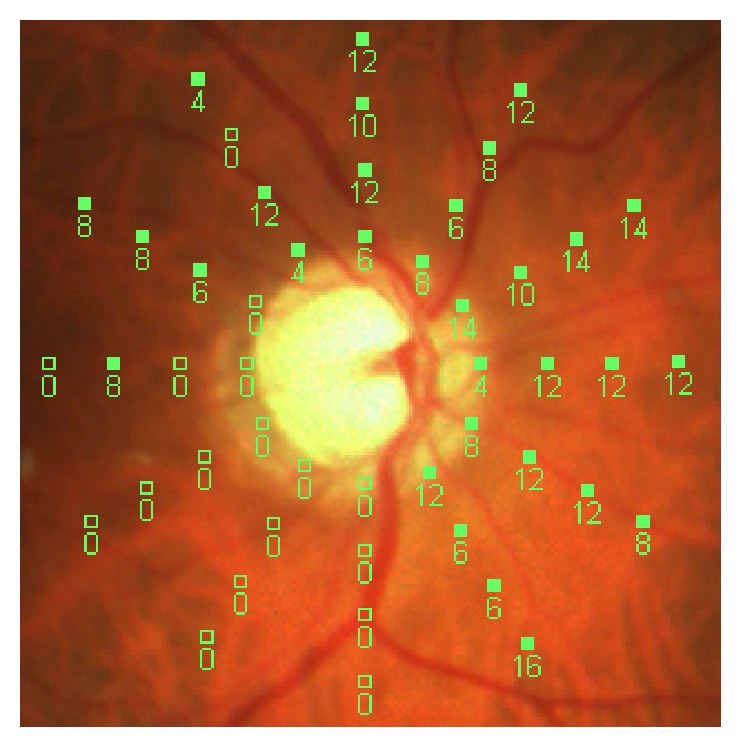
Analysis of the peripapillary retinal sensitivity by Mp-1 projecting light stimuli in radial manner to the optic papillae (12 meridians).

Currently, the use of microperimetry to study glaucoma progression consists in the analysis of peripapillary retinal sensitivity through the use of light stimuli projected radially to the optic disc (12 meridians) (Figure 2).

In this way, microperimetry could be able to detect and monitor the damage of RNFL in subjects affected by glaucoma.

The possibility of evaluating contemporarily both retinal sensitivity and the direct observation of ocular fundus allows limiting microperimetric analysis to selected areas that would show defects localized within the retinal nerve fiber layer (RNFL), leading to the identification of functionally abnormal areas and allowing an eventual follow-up evaluation. In addition, microperimetry is able to detect and monitor precocious and specific RNFL impairment and, when it is conspicuous, could lead to a progressive enlargement of the physiological optic papilla excavation and visual field impairment. Therefore, the early evaluation of morphofunctional alterations at this level is crucial in the diagnosis and prevention of the glaucomatous disease.

**Figure 3:**
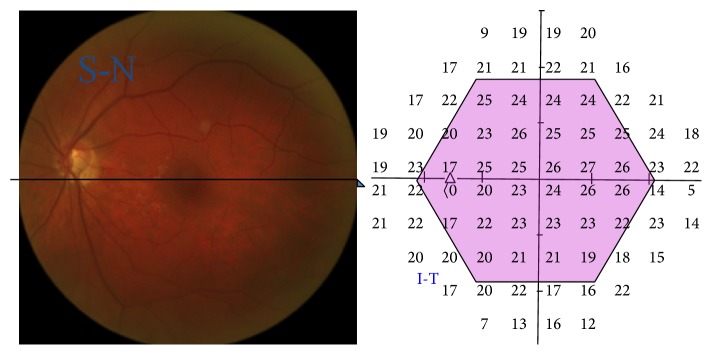
Quadrant correspondence between Mp-1 and Humphrey.

**Figure 4:**
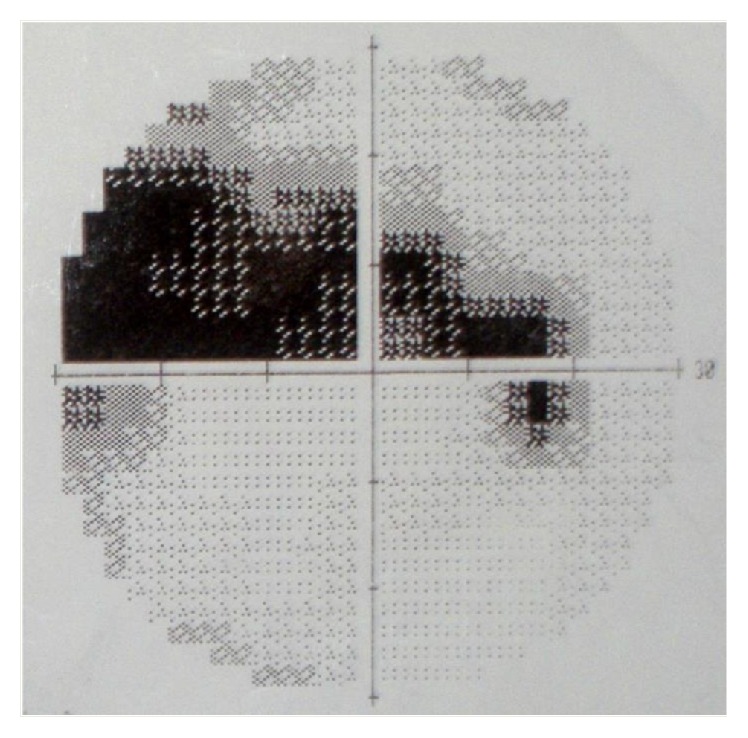
Example of the perimetric abnormalities analyzed by microperimetric test with both Mp-1 (interpolated map) and Humphrey 30-2 perimetry.

The aim of the study was the application of microperimetry, correlating it with Humphrey perimetry 30-2, in patients with primary open-angle glaucoma with perimetric defects “localized” at the papillary level in order to get an assessment of the accuracy of the results obtained by Mp, which could then be used routinely for early diagnosis of the disease and close follow-up in the prevention of damage caused by glaucoma (Figures 3 and 4).

## 2. Materials and Methods

In this study, 40 eyes from 25 patients (10 females and 15 males) affected by chronic open-angle glaucoma or focal papillary perimetric abnormalities, comparing them with Humphrey 30-2 perimetry threshold test, have been included. The exclusion criteria were represented by (i) the opacity of dioptric lens, (ii) previous ocular surgery (cataract, glaucoma, and retinal detachment), and the presence of type II diabetes, blood hypertension, and other systemic disorders.

Each patient underwent an ophthalmologic visit including Best Corrected Visual Acuity (BCVA), anterior segment examination, fundus examinations (evaluation of glaucomatous optic disc damage compared to the value of cup/disc ratio), and gonioscopy to classify anterior chamber angles according to Shaffer system. Furthermore, the same operator performed a tonometric curve during the same day to each patient.

Each patient was tested with microperimetry Nidek Mp-1 (NAVIS software version 1.7.2; Nidek Technologies) applied to conventional static perimetric analysis.

The hardware is composed of an IR fundus camera (IR light with filtered halogen lamp, 768 × 576 pixel resolution) allowing both a dynamic visualization and a real-time retinal examination for a 45° exam angle.

The fundus camera is equipped with a lens system for correcting patient's refractive defect in a range of −12.5/+16 diopters; because of this system the invasiveness and patient's discomfort are reduced during the exam, and the accuracy functional evaluation and focus of the retinal image are improved.

In addition to that, this tool is equipped with a color* fundus camera* (CCD camera* progressive scan*, 780 × 580 pixel resolution, including Xenon flash) allowing the acquisition of high-quality digital retinoic picture where it is possible to select retinal spots (e.g., superficial retinal vessels or optic disc) and a system of* eye-tracking* which is able to compensate instinctive ocular movements occurring during test performance allowing testing the fixation throughout the functional test.

All the tests were performed in the same room with low luminance, by the same skilled operator, minimizing potential biases.

Additional parameters selected for microperimetry included 15 × 15° field, 1.27 cd/m^2^-white background, magnitude of the stimulus Goldmann III white-100 ms, initial attenuation of 20 dB, threshold strategy (4-2-1), 77 tested dots, and single cross fixation sight 1° (Table 1).

Moreover, the computerized campimetric evaluation by Humphrey 30-2 was performed using the following parameters: 15 × 15° field, 1.27 cd/m^2^-white background, magnitude of the stimulus Goldmann III white-100 ms, threshold strategy SITA-Standard, 25 tested dots, and single cross central fixation sight 1° (Table 1). For each campimetric test (H) an inverse subdivision in quadrants was obtained compared to the microperimetric exam (M).

Mean values of dB sensitivity in each tested dot obtained with the microperimetric test were correlated with the corresponding values obtained by conventional static perimetric analysis.

SPSS programme has been used for statistical analysis. Data were analyzed using both Pearson's correlation and Bland-Altman analysis. Data passing *p* < 0.01 cut-off were considered statistically significant.

## 3. Results

Mean age of study population was 56.96 years ± 9.02 (M ± SD; age ranging from 37 to 70 years). All patients showed BCVA values >8/10 (Snellen optotype); slit-lamp exam of anterior chamber was normal and cup/disc ratio was <0.3 tested by direct ophthalmoscopy.

Gonioscopy performed on all subjects detected a 4° angle according to Shaffer.

IOP after tonometric curves resulted in no change during combined therapy with more than one antiglaucoma drug, with mean value of 14 mmHg ± 3 mmHg.

Interpolated results showed mean value of sensitivity for each tested dot with Mp-1 of 11.98 dB ± SD 4.31. They may be correlated with mean total values or with mean values of the corresponding quadrant tested by Humphrey 30-2 perimetry (17.95 dB with SD 4.32), with correlation coefficient of 0.556 (Table 2(a)).

The comparison for corresponding quadrants with regard to the defects observed resulted to be statistically significant for parapapillary sectors, with values of H3-M3 (H3-11.06 dB; M3-13.50 dB) and H4-M4 (H4-18.17 dB; M4-11.77 dB), with 0.762 and 0.456 (Table 2(b)), respectively.

**Figure 5:**
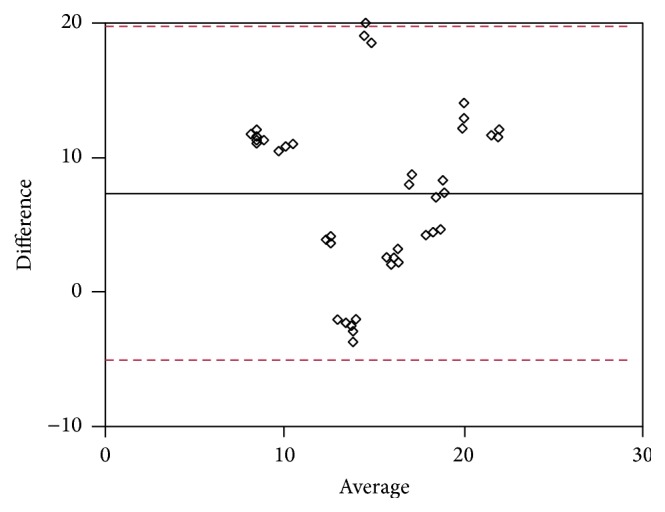


**Figure 6:**
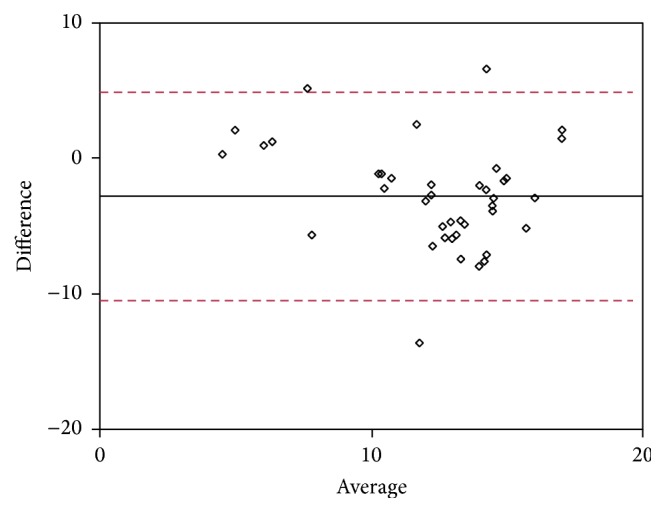


**Figure 7:**
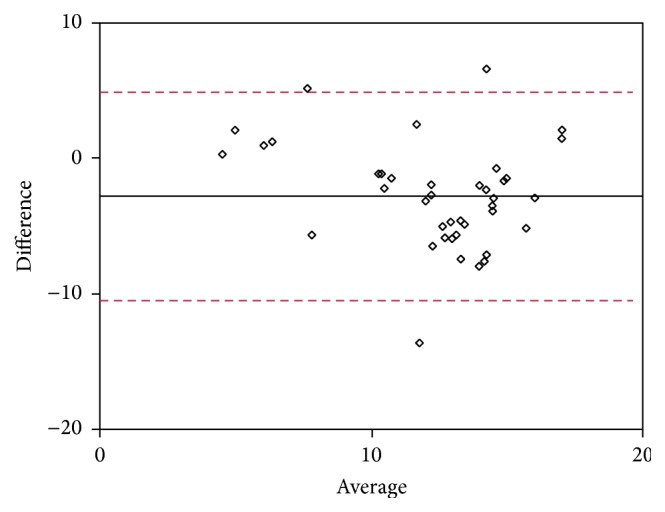


**Figure 8:**
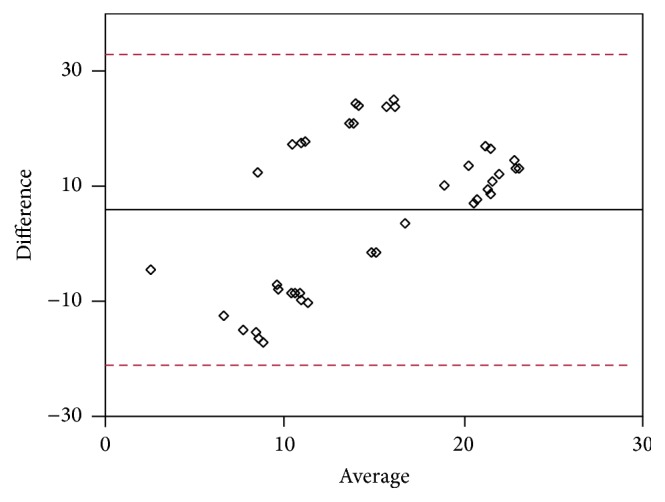


Data were obtained by Bland-Altman analysis. As shown in Tables 3 (Figure 5), 4 (Figure 6), 5 (Figure 7), and 6 (Figure 8), there was an average value of 7.35 (SD: 6.17) for S-T/I-N (H1-M1) quadrants, an average value of 12.24 (SD: 5.245) for I-T/S-N (H2-M2) quadrants, an average value of −2.802 (SD: 3.836) for I-N/S-T (H3-M3) quadrants, and an average value of 5.822 (SD: 13.48) for S-N/I-T (H4-M4) quadrants.

## 4. Discussion

In the last years, microperimetry has been widely used in case of macular disorders, evaluating retinal sensitivity of central area [2, 3]. Its application in glaucomatous optic neuropathy is recent with different modalities for various analyses. The acquisition of a new-generation microperimetric method is better than previous tests that lacked standardization of the procedure; it was impossible for them to perform follow-up tests for the same retinal spot, and they lacked colored visualization of the fundus. This technical improvement led to an increased interest for its application on glaucoma. Because of these advantages Mp-1 has been used to evaluate functional and morphological alterations at molecular level and the fixation in glaucoma-affected patients [4, 5]. To our knowledge, no previous studies evaluated the correlation between Mp-1 and Humphrey 30-2 perimetry outcomes for the evaluation of papillary-derived view field defects. Up to now,* Humphrey 30-2* perimetry is considered the gold standard for the diagnosis and follow-up of glaucomatous diseases. The analyses of our outcomes led to considering that conventional perimetry using Mp-1 microperimeter in patients affected by chronic glaucoma is accurate because of the statistically significant correlation with Humphrey 30-2 perimetry.

This correlation is even more marked for the campimetric inferonasal quadrant (microperimetric superotemporal quadrant) and superonasal quadrant (microperimetric inferotemporal quadrant), well known to be primarily affected by loss of ganglion cells.

The relevance of obtained results is related to the accuracy of this new analysis in evaluating functional glaucoma damage. Furthermore, it is a morphofunctional procedure that is able to evaluate the sensitivity threshold with topographic accuracy, as it is possible to establish with accuracy the retinic dots that are stimulated by a system of direct retinal visualization.

This aspect is relevant because Mp-1 would result as a follow-up test, highly useful to monitor glaucoma. The traditional electronic Humphrey 30-2 perimetry consists in identifying stimulated retinic areas in their geometric position compared to the patient's fixation point. This type of test does not provide any correlation between ocular fundus test and the retinal sensitivity in a specific point, as it is not possible to have a direct observation of ocular fundus and to identify where the light stimuli will be projected.

Topographic visualization of the perimetric defect detected by microperimetry would allow retesting zones with reduced sensitivity (localized abnormalities) which are topographically visualized and displayable on the ocular fundi, avoiding worsening of the functional defect by a better modulation of the antiglaucoma therapy and allowing better monitoring of the pathologic functional damage.

Therefore, we may conclude that Mp-1 microperimetry is a sensitive method that provides detailed topographic maps easy to read and could be used for glaucomatous patient's follow-up correlated with methods that are widely standardized in the glaucoma semeiotic.

## Figures and Tables

**Table 1:** Parameters used with both Mp-1 and Humphrey perimetry methods.

Parameter	Humphrey	Mp-1
Field	15° × 15°	15° × 15°

Background	31.3-white	1.27 cd/m^2^-white

Stimuli	Goldmann III	Goldmann III
	White-100 ms	White-100 ms

Strategy	SITA-Standard	4-2-1

Fixation sight	Central	Single cross 1°

Tested dots	76	77

**(a):** 

Pearson's correlation results			
	Mean sensitivity (dB)	Std. deviation	Correlation coefficient
H1	18.60	5.15	0.297
M1	11.24	5.13	

H2	23.32	4.03	0.295
M2	10.68	3.20	

H3	11.06	3.25	0.762
M3	13.50	4.23	

H4	18.17	10.84	0.456
M4	11.77	5.87	

H tot	17.95	4.32	0.556
M tot	11.98	4.31	

H, Humphrey 30-2; M, Mp-1.

**(b):** 

Pearson's correlations								
	H1 S-T	M1 I-N	H2 I-T	M2 S-N	H3 I-N	M3 S-T	H4 S-N	M4 I-T
H1 S-T								

M1 I-N	0.297^*∗∗*^							

H2 I-T								

M2 S-N			0.295^*∗∗*^					

H3 I-N								

M3 S-T					0.762^*∗∗*^			

H4 S-N								

M4 I-T							0.456^*∗∗*^	

^*∗∗*^Correlation is significant at the 0.01 level (2–4 tailed).

**Table 3:** Quarter S-N/I-T Bland-Altman analysis (see Figure 5).

Method 1	Method 2	Bland-Altman analysis	
		Difference	Average
12.4	15.3	−2.9	13.85
26.5	13.6	12.9	20.05
27.7	16.2	11.5	21.95
15.5	4.7	10.8	10.1
22.6	15.3	7.3	18.95
24.1	5.6	18.5	14.85
14.1	2.8	11.3	8.45
20.5	16.1	4.4	18.3
14.3	2.7	11.6	8.5
12.4	14.6	−2.2	13.5
17.5	15.3	2.2	16.4
14.5	10.8	3.7	12.65
17.4	14.9	2.5	16.15
21.5	12.8	8.7	17.15
12.5	15	−2.5	13.75
27	13	14	20
28	16	12	22
16	5	11	10.5
22	15	7	18.5
24	5	19	14.5
14	3	11	8.5
20	15.8	4.2	17.9
14.5	2.5	12	8.5
12	14	−2	13
17	15	2	16
14.7	10.6	4.1	12.65
17	14.5	2.5	15.75
21	13	8	17
12	15.7	−3.7	13.85
26	13.9	12.1	19.95
27.4	15.8	11.6	21.6
15	4.5	10.5	9.75
23	14.7	8.3	18.85
24.5	4.6	19.9	14.55
14.5	3.2	11.3	8.85
21	16.4	4.6	18.7
14	2.3	11.7	8.15
13	15	−2	14
18	14.8	3.2	16.4
14.3	10.4	3.9	12.35

	Average	7.35	
	SD	6.171	

**Table 4:** Quarter I-T/S-N Bland-Altman analysis (see Figure 6).

Method 1	Method 2	Bland-Altman analysis	
		Difference	Average
25.4	10.61	14.79	18
28	10.4	17.6	19.2
25.8	12.7	13.1	19.25
20.1	6.5	13.6	13.3
18.9	17.7	1.2	18.3
20.3	4.7	15.6	12.5
25.9	9.7	16.2	17.8
23.5	13.1	10.4	18.3
27.6	9.8	17.8	18.7
15	10.7	4.3	12.85
27.5	14.2	13.3	20.85
26	9.4	16.6	17.7
25	14.2	10.8	19.6
27.5	11.2	16.3	19.35
25.5	10	15.5	17.75
20.4	10.2	10.2	15.3
19.2	13	6.2	16.1
20	6	14	13
23	17.5	5.5	20.25
26.2	5	21.2	15.6
23	10	13	16.5
25.2	13	12.2	19.1
28	10.1	17.9	19.05
14.7	10.4	4.3	12.55
26	14	12	20
28.5	9	19.5	18.75
26.1	11	15.1	18.55
19.8	10.8	9	15.3
18.6	12.5	6.1	15.55
19.7	7	12.7	13.35
22.7	18	4.7	20.35
25.6	4.5	21.1	15.05
24	9.5	14.5	16.75
25.6	11	14.6	18.3
27.5	14.5	13	21
15.2	9.7	5.5	12.45
15.5	10.3	5.2	12.9
27.2	9.6	17.6	18.4
25.2	10.6	14.6	17.9
26	23.2	2.8	24.6

	Average	12.24	
	SD	5.245	

**Table 5:** Quarter I-N/S-T Bland-Altman analysis (see Figure 7).

Method 1	Method 2	Bland-Altman analysis	
		Difference	Average
10.6	15.3	−4.7	12.95
10.4	13.6	−3.2	12
12.7	16.2	−3.5	14.45
6.5	5.6	0.9	6.05
17.7	16.3	1.4	17
4.7	4.4	0.3	4.55
9.7	10.9	−1.2	10.3
13.1	18.3	−5.2	15.7
9.8	11	−1.2	10.4
13.1	15.4	−2.3	14.25
9.8	15.7	−5.9	12.75
10.7	17.8	−7.1	14.25
14.2	15.7	−1.5	14.95
9.4	11.6	−2.2	10.5
14.2	15	−0.8	14.6
11.2	13.2	−2	12.2
10	16	−6	13
10.2	5.2	5	7.7
13	16	−3	14.5
6	4	2	5
17.5	11	6.5	14.25
5	18.6	−13.6	11.8
10	11.5	−1.5	10.75
13	15	−2	14
10.1	15.2	−5.1	12.65
10.4	18	−7.6	14.2
14	15.7	−1.7	14.85
9	15.5	−6.5	12.25
11	15.6	−4.6	13.3
10.8	13.6	−2.8	12.2
12.5	16.4	−3.9	14.45
7	5.8	1.2	6.4
18	16	2	17
5	10.7	−5.7	7.85
10	18	−8	14
12.9	10.5	2.4	11.7
10.3	16	−5.7	13.15
11	15.9	−4.9	13.45
14.5	17.5	−3	16
9.6	17	−7.4	13.3

	Average	−2.802	
	SD	3.836	

**Table 6:** Quarter S-N/I-T Bland-Altman analysis (see Figure 8).

Method 1	Method 2	Bland-Altman analysis	
		Difference	Average
0.3	15.3	−15	7.8
27	13.6	13.4	20.3
0.7	16.2	−15.5	8.45
28	4.4	23.6	16.2
14.3	16	−1.7	15.15
24.2	3.6	20.6	13.9
19.6	2.3	17.3	10.95
6.2	16.6	−10.4	11.4
26.1	2.2	23.9	14.15
6.3	15	−8.7	10.65
0.3	16.9	−16.6	8.6
29.7	13.4	16.3	21.55
24	17.1	6.9	20.55
29.6	12.8	16.8	21.2
18.5	15	3.5	16.75
0.4	13	−12.6	6.7
28	16	12	22
27.5	4	23.5	15.75
14	15.8	−1.8	14.9
24	3.3	20.7	13.65
19	2	17	10.5
6	16	−10	11
26	2	24	14
6	14.8	−8.8	10.4
26	16.7	9.3	21.35
6	13.3	−7.3	9.65
0.2	17.5	−17.3	8.85
30	15.7	14.3	22.85
23.9	14	9.9	18.95
29.4	16.5	12.9	22.95
0.3	5	−4.7	2.65
27	16.2	10.8	21.6
28.5	3.8	24.7	16.15
14.6	2.5	12.1	8.55
24.5	17	7.5	20.75
20	2.4	17.6	11.2
6.5	15.3	−8.8	10.9
25.8	17.2	8.6	21.5
5.7	13.7	−8	9.7
29.5	16.6	12.9	23.05

	Average	5.822	
	SD	13.48	
